# How Actin Filaments Doff Their Pi Cap

**DOI:** 10.1371/journal.pbio.1001163

**Published:** 2011-09-27

**Authors:** Caitlin Sedwick

**Affiliations:** Freelance Science Writer, San Diego, California, United States of America

**Figure:**
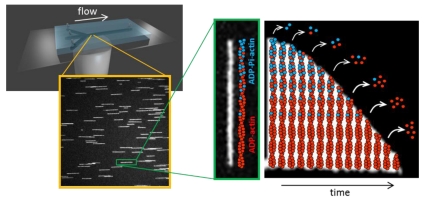
Actin filaments are aligned by a microfluidic flow, and observed with light microscopy. Their depolymerization reveals their composition, as ADP-actin subunits dissociate faster than ADP-Pi-actin subunits. Image credits: Antoine Jégou and Guillaume Romet-Lemonne.

The cytoskeletal protein actin (or one of its relatives) is found in almost all life forms. Yet, despite its ubiquity, there are still some fundamental things about its structure and function that are not well understood. For example, we know that monomeric actin subunits (called G-actin) polymerize into filaments (F-actin), but it's still unclear how actin dynamics–filament assembly and disassembly, and turnover of individual actin subunits–are managed within these filaments. As cellular processes like motility are regulated by actin polymerization and depolymerization, understanding actin dynamics will provide important insight into these biological processes and how they in turn are regulated. Toward that end, Antoine Jégou and colleagues examine the details of actin filament polymerization and depolymerization in their paper published this week in *PLoS Biology*.

It's well known that individual actin molecules bind to the nucleotide ATP, an energy-providing molecule. Actin filaments grow asymmetrically by adding ATP-actin to just one end of the filament (called the “barbed end”; the opposite end is called the “pointed end”). ATP-actin addition is followed rapidly by ATP hydrolysis, leaving behind ADP plus a phosphate molecule (called Pi) bound to the actin subunit within the filament. These ADP-Pi-actin subunits can then release Pi to generate ADP-actin, and the subunits can eventually disassemble. Thus, a growing actin filament is composed of three different species of actin: ATP-actin (at the very tip), ADP-Pi-actin, and ADP-actin. But many proteins that interact with actin have different affinities for these different forms of actin, and actin polymerization can be regulated by proteins binding to the filaments, making it a challenge to understand how these different species contribute to the makeup of actin filaments. Jégou and colleagues were particularly interested to know more about how Pi is released from ADP-Pi-actin within actin filaments, as this destabilizes the actin-actin bonds and thus lowers the filament rigidity.

There are two theories that may explain what controls Pi release in actin filaments. The “vectorial” hypothesis holds that Pi release can only happen on ADP-Pi-actin subunits that abut an ADP-actin subunit, while the “random” hypothesis says that Pi release can happen anywhere in the actin filament. Jégou et al. set out to test these two hypotheses.

Unfortunately, you can't tell the nucleotide composition of a given actin subunit just by looking at it. Instead, it's necessary to infer this information by watching how the filament as a whole behaves–specifically, how it disassembles. Actin filaments are polymers in equilibrium with monomers, and taking the monomers away causes their depolymerization. Adding additional free ATP-actin to the environment causes filaments to elongate at their barbed ends; taking it away makes them shrink from the barbed end on back. But ADP-Pi-actin falls off the barbed end of a filament more slowly than does ADP-actin, so, by removing all the free actin and watching the rate at which filaments dissolve, one can infer whether the actin coming off the barbed end is ADP-actin or ADP-Pi-actin. Jégou and colleagues designed a special apparatus to carry out these observations.

The new apparatus uses microfluidics to quickly remove all the free actin from a chamber containing actin filaments (that are anchored by their pointed ends to the chamber walls). As individual filaments then shrink in length, their behavior can be monitored with a microscope. Using this setup, the authors saw that filaments start out shrinking rather slowly, but that the rate of shrinkage accelerates in an exponential fashion.

The only way to account for this behavior, say Jégou et al., is if Pi release from actin is random, not vectorial. They explain that actin subunits closer to the pointed end have been part of the filament for longer, and have had a long time to release their Pi and become ADP-actin, which falls off quickly. Those subunits closest to the barbed end, however, are likely to still be ADP-Pi-actin, which fall off more slowly. Therefore, as a filament shrinks, it first works its way through a cap of slowly dissociating ADP-Pi-actin, then gradually starts encountering higher frequencies of faster-dissociating ADP-actin, so that the overall disassembly rate accelerates in exponential fashion. If Pi dissociation were vectorial, one would have expected to observe an abrupt change in depolymerization rate.

With this result in hand, Jégou and colleagues next turned their attention to an actin-binding protein called profilin, which is known to play an important role in controlling actin filament assembly. Profilin, they showed, accelerates actin filament disassembly. It does this not by changing the rate at which Pi is released from actin, which would have the effect of increasing the amount of fast-dissociating ADP-actin in the filament. Instead, it speeds the rate at which both ADP-Pi-actin and ADP-actin fall off the end of a filament.

Jégou and colleagues' observations also have implications for understanding how profilin controls the addition of actin monomers to the growing barbed end of actin filaments. Future experiments will expand on this aspect of their work, but in the meantime, these results resolve controversies about both Pi release and profilin's role that have been brewing for many years, and offer a clearer picture of the underlying dynamics of the actin filament.

**Jégou A, Niedermayer T, Orbán J, Didry D, Lipowsky R, et al. (2011) Individual Actin Filaments in a Microfluidic Flow Reveal the Mechanism of ATP Hydrolysis and Give Insight into the Properties of Profilin. doi:10.1371/journal.pbio.1001161**

